# Prevalence and risk factors of primary sarcopenia in community-dwelling outpatient elderly: a cross-sectional study

**DOI:** 10.1038/s41598-020-75250-y

**Published:** 2020-11-11

**Authors:** Visaratana Therakomen, Aisawan Petchlorlian, Narisorn Lakananurak

**Affiliations:** 1 Department of Medicine, Faculty of Medicine, Chulalongkorn University, King Chulalongkorn Memorial Hospital, The Thai Red Cross Society, Bangkok, Thailand; 2 Division of Geriatric Medicine, Department of Medicine, Faculty of Medicine, Chulalongkorn University, King Chulalongkorn Memorial Hospital, The Thai Red Cross Society, Bangkok, Thailand; 3 Division of Clinical Nutrition, Department of Medicine, Faculty of Medicine, Chulalongkorn University, King Chulalongkorn Memorial Hospital, The Thai Red Cross Society, 254 Phayathai Road, Pathumwan, Bangkok, 10330 Thailand

**Keywords:** Nutrition disorders, Geriatrics, Risk factors

## Abstract

No previous study has investigated the prevalence and risk factors for primary sarcopenia in outpatient setting. This study aims to evaluate the prevalence and factors associated with primary sarcopenia in outpatient elderly. Additionally, we compared the severity of sarcopenia based on the 2014 and 2019 Asian Working Group for Sarcopenia (AWGS) criteria. This cross-sectional study was performed in 330 subjects aged over 60 years in an outpatient setting. The muscle strength, muscle performance and muscle mass were assessed using the handheld dynamometer, 6-m gait speed, and bioelectrical impedance analysis, respectively. The prevalence of sarcopenia was 10% as per the 2014 and 2019 AWGS criteria. The development of sarcopenia was positively correlated with the age with an odds ratio (OR) of 6.87 [95% confidence interval (CI) 1.63–28.88] in the middle-old group (70–79 years), and 13.71 (95%CI 3.66–51.41; *p* = 0.009) in the very old group (≥ 80 years). Prefrailty and low physical activity were significantly associated with sarcopenia with an OR of 4.75 (95%CI 1.90—11.89) in prefrailty, 15.35 (95%CI 1.69–139.47) in the middle activity group, and 17.99 (95%CI 1.95–165.73) in the lowest activity group. In conclusion, primary sarcopenia was found in one-tenth of outpatient elderly. Age, prefrailty, and low activity were independent factors associated with sarcopenia.

## Introduction

Sarcopenia is defined as a generalized and progressive loss of skeletal muscle mass and muscle function (muscle strength and/or physical performance)^1,2^. It has been accepted as one of the geriatric syndromes and is one of the most important public health concerns since it can result in functional decline, physical disability, falling, increased hospitalization and health care cost, poor quality of life, and death^3^. Moreover, sarcopenia can occur in obese elderly, which the co-occurrence of these two conditions is known as sarcopenic obesity. The pathogenesis is complex and involves age-related changes in muscle mass and hormone, inflammation, and insulin resistance^4^. Sarcopenic obesity is associated with an increased risk of physical disability, cardiovascular diseases, hospitalization, and mortality compared to obese or sarcopenic elderly alone^5,6^.

Sarcopenia can be divided into primary or age-related sarcopenia and secondary sarcopenia. Primary sarcopenia is diagnosed when no other specific cause is evident. It is associated with mitochondrial dysfunction, satellite cells dysfunction, neuromuscular dysfunction (α-motoneuron degeneration and muscle fibre denervation), reduction in anabolic hormone productions or sensitivity, and anorexia of aging^7^. On the other hand, secondary sarcopenia is considered when factors other than aging are evident, especially systemic diseases such as malignancy or organ failure. Therefore, appropriate treatment of secondary causes is imperative to mitigate secondary sarcopenia. The etiology and management of sarcopenia are different among the elderly. Thus, by categorizing sarcopenia into primary sarcopenia and secondary sarcopenia are useful to prevent and treat this condition^8,9^.

The prevalence of sarcopenia varies between studies depending on ethnicity, population setting, and diagnostic criteria. As a result of this, it is difficult to compare the prevalence of sarcopenia between studies. The Asian Working Group for Sarcopenia (AWGS) has proposed practical consensus diagnostic criteria for sarcopenia in 2014, and the criteria were recently revised and updated in 2019^1,10^. The use of such accepted criteria can help correct the confusion in the prevalence of sarcopenia. In regards to the population setting, previous studies that used the 2014 AWGS criteria have reported that the prevalence of sarcopenia was 1–30% in a community setting, 14–33% in a long-term care setting, and up to 60% in in-hospital rehabilitation unit^11–13^. However, sarcopenia in outpatient setting has been evaluated in a few studies and the prevalence ranged from 0.8 to 26%^14–17^. Elders in outpatient setting are usually frailer than those from the community setting. Early detection and treatment of sarcopenia in this population may help prevent hospital admission and reduce health care expenditures.

In order to effectively prevent the development of sarcopenia, it is important to know which risk factors contribute to the condition. Many factors have been identified to contribute to sarcopenia such as age, sex, physical activity, falls, frailty, comorbidities, body mass index (BMI), dietary intake, and malnutrition, however; this was not consistent across all studies^13,18–20^. One of the reasons is that most of the previous studies did not clearly differentiate between primary and secondary sarcopenia. Risk factors associated with sarcopenia are different between primary and secondary sarcopenia. Multiple risk factors may contribute to the development of primary sarcopenia whereas secondary sarcopenia is usually associated with only few obvious causes^3^. Although the categories of primary and secondary sarcopenia may be beneficial for risk factor identification, differentiation between primary and secondary sarcopenia cannot be easily done in some situations such as elder patients with multiple comorbidities.

The aim of this study is to describe the prevalence of primary sarcopenia in outpatient setting. The prevalence of possible sarcopenia, sarcopenia, and severe sarcopenia as per the 2014 AWGS criteria was compared to the 2019 AWGS criteria. The risk factors associated with sarcopenia were also comprehensively evaluated.

## Materials and methods

### Patients

This is a cross-sectional study that enrolled people older than 60 years old who attended the outpatient clinic at the King Chulalongkorn Memorial Hospital, Bangkok, Thailand, between October 2017 and July 2018. We only included participants who were able to walk. Patients with potential secondary causes of sarcopenia such as active malignancy, chronic heart failure, chronic kidney diseases stage 4 or 5, cirrhosis, chronic obstructive pulmonary disease, autoimmune diseases, hyperthyroid, stroke, parkinsonism, uncontrolled diabetes mellitus with complications, and chronic use of steroid were excluded from the study. Also, patients with factors that affected the bioelectrical impedance analysis (BIA) measurement, including fluid overload and limb amputation, were excluded from the study. The participants were divided into three groups according to their age: the young-old group (age 60–69 years), the middle-old group (age 70–79 years), and the very old group (age ≥ 80 years).

This study was approved by the Institutional Review Board of the Faculty of Medicine, Chulalongkorn University, Bangkok, Thailand (IRB number 098/60). We approached all patients older than 60 years old and provided them with the information about the study. Written informed consent was obtained from the participants before any procedures were carried out. All methods were carried out in accordance with relevant guidelines and regulations.

### Sarcopenia determination

Sarcopenia was diagnosed according to the diagnostic algorithm of the 2014 AWGS criteria^1^. Handgrip strength and gait speed were used as screening tools. Handgrip strength was done using the hand-held dynamometer (Jamar hand dynamometer, Preston Jackson, Michigan, 49203, USA). Participants were asked to squeeze the dynamometer using their dominant hand as strong as they could, adducted arm beside the body and the elbow was flexed to a 90° angle. Handgrip strength was tested two times and the best value was selected. The cut-off value for handgrip strength for male was 26 kg and for female was 18 kg. Gait speed was evaluated by requesting the participants to walk a distance of 8 m. Timer was started at the point of 1-m distance and stopped at 7-m distance to get a total range of 6 m. This was done twice and the best value was recorded with the cut-off value of 0.8 m/s. Elderly who had handgrip strength and/or gait speed lower than the cut-off values were sent to measure their muscle mass by multifrequency BIA (InBody model 720). Participants were asked to wear normal indoor clothing and advised to stand barefooted in upright position with their feet on the feet electrodes on the machine platform and their arms abducted with hands gripping on to the hands electrodes on the handles. The cut-off value for low muscle mass in male was 7 kg/m^2^ and in female was 5.7 kg/m^2^.

The prevalence of sarcopenia was also calculated by using the 2019 AWGS criteria. The updated criteria retained the requirement for evaluating the muscle strength, physical performance, and skeletal muscle mass to identify sarcopenia. However, the cut-off value for low muscle strength by the handgrip strength measurement was changed to < 28 kg for men, and the speed for the 6-m walk < 1 m/s indicated low physical performance. The criteria also categorized sarcopenia according to the severity of the condition: possible sarcopenia, sarcopenia, and severe sarcopenia. Possible sarcopenia was defined as having either low muscle strength or low physical performance. Sarcopenia was defined as having low muscle mass and low muscle strength or low physical performance. Severe sarcopenia was defined as having low muscle mass, low muscle strength, and low physical performance^10^.

Sarcopenic obesity was defined as sarcopenic patients having BMI ≥ 25 kg/m^2^, according to World Health Organization (WHO)-Asian BMI-classification.

### Data collection

Demographic data including age, sex, body weight, height, BMI, and comorbidities were recorded. Age, sex, BMI, frailty, physical activity, fall history, osteoarthritis of the knee, depression, Charlson comorbidity index, malnutrition, and protein intake were analyzed as potential risk factors for sarcopenia. Frailty was evaluated by using Fried frailty phenotype assessment^21^. Physical activity in 1 week was recorded, analyzed, and subcategorized into the lowest group (< 33rd percentile), the moderate group (33rd–66th percentile), and the highest group (> 66th percentile). Fall history was acquired by asking participants the number of falls in the past 1 year. An experienced dietitian made the diagnosis of malnutrition by using the Mini Nutritional Assessment (MNA) and assessed protein intake using a 3-day food record.

### Statistical analysis

Based on a previous study^13^, the prevalence of sarcopenia in Thai community-dwelling elderly was 30.5%. Thus, the required sample for an α = 0.05 and Z = 1.96 was estimated to be 326 participants.

Data were analyzed using SPSS Statistics version 18 (SPSS, Inc., Chicago, IL, USA). Differences in demographic data, frailty, physical activity, fall history, osteoarthritis of the knee, depression, malnutrition, and protein intake between the elderly with and without sarcopenia were analyzed by Pearson’s chi-square for categorical parameters, independent samples t-test for normally distributed continuous data, and Mann–Whitney U test for non-normally distributed continuous data. Risk factors for sarcopenia were evaluated and identified using simple regression analysis and multiple regression analysis. Data were shown as number, number and percentage, mean ± standard deviation, median and range, or median and interquartile range. A *p* value of less than 0.05 was regarded as being statistically significant.

## Results

A total of 330 elder participants were enrolled into the study and the mean age (SD) was 66.89 (5.51) years. Three-fourths of the patients were between 60 and 69 years of age. There were 270 females (81.8%) and 60 males (18.2%). The mean BMI (SD) was 23.08 (3.56) kg/m^2^. Around fifty percent of the participants had BMI within the normal range (18.5–22.9 kg/m^2^). Overweight and obesity were found in 50 cases (15.1%) and 89 cases (27.0%), respectively. The two most common comorbidities were dyslipidemia (30.6%) and hypertension (25.2%). Many of the participants had Charlson comorbidity index of zero (85.8%). The demographic characteristics of the study population are summarized in Table 1.

**Table 1:** Demographic data of all study participants.

Demographic data	n = 330
Age, mean ± SD (years)	66.89 ± 5.51
Age, n (%)	
60–69 years	248 (75.2%)
70–79 years	67 (20.3%)
≥ 80 years	15 (4.5%)
Sex, n (%)	
Male	60 (18.2%)
Female	270 (81.8%)
Body weight, mean ± SD (kg)	56.27 (10.40)
Height, mean ± SD (cm)	155.86 (6.88)
Body mass index, mean ± SD (kg/m^2^)	23.08 (2.56)
Body mass index, n (%)	
< 18.5 kg/m^2^	32 (9.7%)
18.5–22.9 kg/m^2^	159 (48.2%)
23–24.9 kg/m^2^	50 (15.1%)
≥ 25 kg/m^2^	89 (27%)
Comorbidities, n (%)	
Dyslipidemia	101 (30.6%)
Osteoarthritis of knee	88 (26.7%)
Hypertension	83 (25.2%)
Diabetes mellitus	27 (8.2%)
Osteopenia/Osteoporosis	16 (4.9%)
Gastroesophageal reflux disease	8 (2.4%)
Coronary artery disease	3 (0.9%)
Charlson comorbidity index, n (%)	
0	283 (85.8%)
1	44 (13.3%)
2	3 (0.9%)

*SD* standard deviation.

### Prevalence of primary sarcopenia

**Figure 1:**
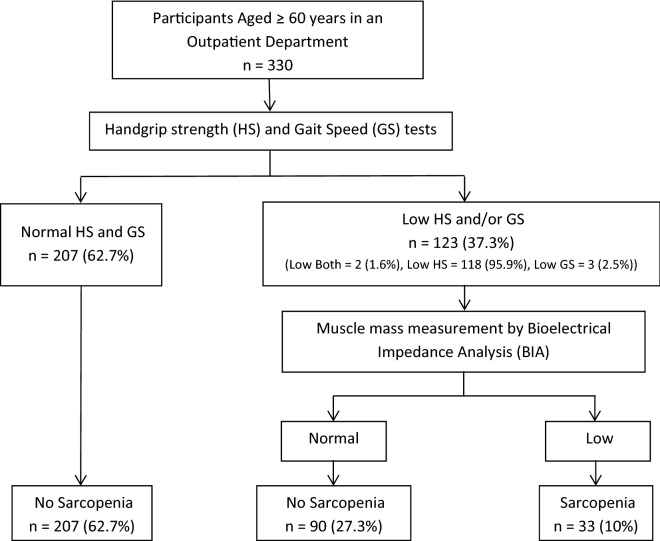
Diagram of the screening method for sarcopenia according to the 2014 Asian Working Group for Sarcopenia (AWGS) criteria and the number of participants at each step.

According to the 2014 AWGS criteria, the overall prevalence of primary sarcopenia in Thai community-dwelling outpatient elderly was 10% (33/330 patients). One hundred and twenty-three subjects (37.3%) had abnormal handgrip strength and/or gait speed. From this group, low grip strength, low gait speed, and low in both tests were found in 118 cases (95.9%), 3 cases (2.5%), and 2 cases (1.6%), respectively. The diagram of the diagnostic method for sarcopenia is shown in Fig. 1. The prevalence of sarcopenic obesity among all of the patients was 1.2% (4/330 patients) and in the obese group, it was 4.5% (4/89 patients).

When the 2019 AWGS criteria were used, the prevalence of sarcopenia and severe sarcopenia were 8.8% (29/330) and 1.2% (4/330), respectively. The possible sarcopenia was identified in 93/330 cases (28.2%). The prevalence of possible sarcopenia, sarcopenia, and severe sarcopenia as per the 2014 AWGS criteria was compared to the 2019 AWGS criteria (Table 2).

**Table 2:** Prevalence of possible sarcopenia, sarcopenia, and severe sarcopenia using the cut-off values from the 2014 and 2019 AWGS criteria.

	The 2014 AWGS criteria (n)	The 2019 AWGS criteria (n)
No sarcopenia	207 (62.7%)	204 (61.8%)
Possible sarcopenia	90 (27.3%)	93 (28.2%)
Sarcopenia	33 (10%)	29 (8.8%)
Severe sarcopenia	0	4 (1.2%)

*AWGS* The Asian Working Group for Sarcopenia.

### Risk factors for sarcopenia

The significant risk factor for sarcopenia was increased age. Sarcopenia was significantly higher among the middle-old group (age 70–79 years) and the very old group (age ≥ 80 years) (*p* < 0.001). Even though there was no patient diagnosed with frailty in this study, the proportion of patients with prefrailty was significantly higher in patients with sarcopenia (66.7% vs. 34%, *p* < 0.001). Only 9% of sarcopenic patients were in the highest physical activity group whereas 45.5% were in the lowest physical activity group. (*p* = 0.005). Other risk factors (sex, BMI, history of fall, depression, osteoarthritis of the knee, Charlson comorbidity index, malnutrition, and protein intake) were not significantly different between those with and without sarcopenia. (Table 3).

**Table 3:** Factors associated with primary sarcopenia in Thai community-dwelling outpatient setting.

Factors	No Sarcopenia (n = 297)	Sarcopenia (n = 33)	*p* value
**Sex, n**			
Male	54 (18.2%)	6 (18.2%)	1
Female	243 (81.8%)	27 (81.8%)	
**Age (years), n**			
60–69	232 (78.1%)	16 (48.5%)	**< 0.001**
70–79	57 (19.2%)	10 (30.3%)	
> 80	8 (2.7%)	7 (21.2%)	
**BMI (kg/m**^**2**^**), n**			
< 18.5	29 (9.8%)	3 (9.1%)	0.206
18.5–22.9	140 (47.1%)	19 (57.6%)	
23–24.9	43 (14.5%)	7 (21.2%)	
≥ 25	85 (28.6%)	4 (12.1%)	
**Frailty, n**			
No frailty	196 (66%)	11 (33.3%)	**< 0.001**
Prefrailty	101 (34%)	22 (66.7%)	
Frailty	0 (0%)	0 (0%)	
**Physical activity**^**a**^**, n**			
Lowest group	95 (32%)	15 (45.5%)	**0.005**
Middle group	96 (32.3%)	15 (45.5%)	
Highest group	106 (35.7%)	3 (9%)	
**History of Fall (time/year), n**			
0	197 (66.3%)	24 (72.7%)	0.475
1	67 (22.6%)	5 (15.2%)	
≥ 2	33 (11.1%)	4 (12.1%)	
**Depression**, n			
No	287 (96.6%)	32 (97%)	0.971
Yes	10 (3.4%)	1 (3%)	
**Osteoarthritis of Knee, n**			
No	217 (73.1%)	25 (75.8%)	0.409
Yes	80 (26.9%)	8 (24.2%)	
**Charlson comorbidity index**			
0	254 (85.5%)	29 (87.9%)	0.910
1	41 (13.8%)	3 (9.1%)	
2	2 (0.7%)	1 (3%)	
**Nutrition status (MNA), n**			
Normal	228 (76.8%)	20 (60.6%)	0.179
At risk of malnutrition	68 (22.9%)	13 (39.4%)	
Malnutrition	1 (0.3%)	0 (0%)	
**Low protein intake** (< 0.8 g/kg/day), n			
No	83 (27.9%)	10 (30.3%)	0.820
Yes	214 (72.1%)	23 (69.7%)	
**Protein intake (g/kg/day), n**			
< 0.6	114 (38.4%)	10 (30.3%)	0.395
0.6–0.79	100 (33.7%)	13 (39.4%)	
0.8–0.99	46 (15.5%)	4 (12.1%)	
≥ 1	37 (12.4%)	6 (18.2%)	

Statistically significant *p*-values < 0.05 are in bold.

*BMI* body mass index, *MNA* mini nutritional assessment.

^a^The lowest group is 1st–33rd percentile: ≤ 1376 kcal/week, the middle group is 34th–66th percentile: 1376–2764 kcal/week, and the highest group is 67th–100th percentile: ≥ 2764 kcal/week.

Data from the multiple logistic regression analysis showed that patients with increasing age had a significantly higher risk for developing sarcopenia, with an odds ratio (OR) of 6.87 [95% Confident Interval (CI) 1.63–28.88; *p* < 0.001] in the middle-old group and an OR of 13.71 (95% CI 3.66–51.41; *p* = 0.009) in the very old group. In addition, prefrailty was also a significant risk factor for sarcopenia with an OR of 4.75 (95% CI 1.90–11.89; *p* = 0.001). As for physical activity, patients with lower physical activity were significantly at a higher risk of developing sarcopenia, with an OR of 15.35 (95% CI 1.69–139.47; *p* = 0.015) in the lowest physical activity group (≤ 1376 kcal/week) and an OR of 17.99 (95% CI 1.95–165.73; *p* = 0.011) in the middle physical activity group (1376–2764 kcal/week). Table 4 shows the OR of factors associated with sarcopenia by the simple and multiple regression analysis.

**Table 4:** Odds ratio of risk factors associated with primary sarcopenia in Thai community-dwelling outpatient setting.

Factors	Simple logistic regression analysis			Multiple logistic regression analysis		
	Odd ratio	95% CI	*p* value	Odd ratio	95% CI	*p* value
**Sex**						
Male	1	–	–			
Female	1.45	0.50–4.12	0.49			
**Age (years)**						
60–69	1	–	–	1	–	–
70–79	2.71	1.13–6.50	**0.025**	6.87	1.63–28.88	**< 0.001**
> 80	14.20	4.30–46.86	**< 0.001**	13.71	3.66–51.41	**0.009**
**BMI (kg/m**^**2**^**)**						
< 18.5	0.76	0.21–2.75	0.678			
18.5–22.9	1	–	–			
23–24.9	1.20	0.47–3.05	0.702			
≥ 25	0.35	0.11–1.05	0.062			
**Frailty**						
No frailty	1	–	–	1	–	–
Prefrailty	5.03	2.12–11.96	** < 0.001**	4.75	1.90–11.89	**0.001**
**Physical activity**^**a**^						
Lowest group	13.50	1.72–105.93	**0.013**	15.35	1.69–139.47	**0.015**
Middle group	13.66	1.74–107.16	**0.013**	17.99	1.95–165.73	**0.011**
Highest group	1	–	–	1	–	–
**History of Fall (time/year)**						
0	1	–	–			
1	0.52	0.17–1.55	0.238			
≥ 2	0.78	0.22–2.73	0.691			
**Osteoarthritis of knee**						
No	1	–	–			
Yes	1.29	0.46–3.60	0.630			
**Charlson comorbidity index**						
0	1	–	–			
1	0.68	0.20–2.35	0.54			
2	4.65	0.41–52.96	0.22			
**Nutrition status (MNA)**						
Normal	1	–	–			
At risk of malnutrition	2.16	0.93–4.95	0.07			
Malnutrition	0.00	–	–			
**Low protein intake** (< 0.8 g/kg/day)						
No	1	–	–			
Yes	1.12	0.43–2.90	0.821			
**Protein intake (g/kg/day)**						
< 0.6	1.40	0.28–6.87	0.682			
0.6–0.79	2.21	0.47–10.47	0.317			
0.8–0.99	1	–	–			
≥ 1	3.19	0.58–17.64	0.184			

Statistically significant *p* values < 0.05 are in bold.

*CI* confidence interval, *BMI* body mass index, *MNA* mini nutritional assessment.

^a^The lowest group is 1st–33rd percentile: ≤ 1376 kcal/week, the middle group is 34th–66th percentile: 1376–2764 kcal/week, and the highest group is 67th–100th percentile: ≥ 2764 kcal/week.

## Discussion

In this study, we investigated the prevalence and risk factors for primary sarcopenia, diagnosed by the AWGS criteria, in Thai community-dwelling outpatient elderly. The prevalence of sarcopenia in our study was 10% by the 2014 and 2019 AWGS criteria which is within the range found in community setting (1–30%)^11,13^ and outpatient setting (0.8–26%)^14–17^. As previously mentioned, the prevalence of sarcopenia varies from study to study. This discrepancy can be explained by the difference in the study population and the diagnostic criteria used to diagnose sarcopenia. In a previous study that was conducted in a community-dwelling Thai elderly, they used the 2014 AWGS criteria to diagnose sarcopenia and reported that the prevalence of sarcopenia was 30.5%^13^. For our study, the prevalence of sarcopenia was lower. This may be due to the lower percentage of the middle-old group (20.30% vs. 35.4%) and the very old group (4.55% vs. 10.3%) in our study, compared to the previous study since age is an evident risk factor for sarcopenia^20^. Moreover, around one-fifth of the patients in previous study were diagnosed with diabetes mellitus. Diabetes mellitus complicated by organ failure was one of the causes of secondary sarcopenia^3^. This possibly explains the lower prevalence of sarcopenia in our study, which the possible causes of secondary sarcopenia were excluded. With regard to outpatient setting, a similar study in Asian population using the AWGS criteria demonstrated that the prevalence of sarcopenia was higher than our study (24%); however, in the previous study, only patients with diabetes mellitus were enrolled, and almost 20% of the participants were diagnosed with chronic kidney disease^16^.

When we used the 2019 revised AWGS criteria, the overall prevalence of sarcopenia was the same as the prevalence obtained from the 2014 criteria (10%). However, the 2019 AWGS criteria were able to detect 4 patients (1.2%) who had severe sarcopenia. In addition, 3 more patients needed to measure skeletal muscle mass by BIA because the 2019 AWGS criteria had a different cut-off value for muscle strength and physical performance. The cut-off value for muscle strength in men was changed from 26 to 28 kg, and the gait speed for a 6-m walk test was changed from 0.8 to 1 m/s. Interestingly, all of these 3 patients had normal skeletal muscle mass which meant that the new cut-off values did not help identify more sarcopenic patients in our study.

Previous reports have identified several key factors that affect the occurrence of sarcopenia. These included age, sex, physical activity, falls, frailty, comorbidities, BMI, protein intake, and malnutrition^13,18–20^. From the multiple logistic regression analysis, we also found that age, prefrailty, and physical activity was associated with primary sarcopenia. Sarcopenia was found in 6.5% of patients aged 60–69 years, 14.9% in patients aged 70–79 years, and 46.7% in patients aged more than 80 years. Many studies have shown that older age was associated with sarcopenia. Evidence demonstrates that progressive loss of muscle mass begins in middle adult and the loss is accelerated after the age of 75 years^11,22^. Decreased anabolic stimuli (e.g. testosterone), sub-clinical level of inflammation, and increased myostatin levels may contribute to muscle mass loss with increased age^23^.

In our study, physical activity less than 400 kcal per day, which is equivalent to moderate physical activity 2 h per day in elderly people weighing 50 kg such as many household chores, cycling, and gardening, was associated with the development of sarcopenia. This corroborated the finding from previous meta-analysis that physical activity was a protective factor against sarcopenia with an OR of 0.45 (95% CI 0.37–0.55)^24^. Therefore, it is important to promote physical activity that is more than 400 kcal per day among the elderly. This may be one of the strategies that can be used to prevent sarcopenia in outpatient elderly.

Another significant risk factor for sarcopenia is prefrailty. Previous study comprising of 273 Japanese community-dwelling older women demonstrated that both prefrailty and frailty were related to sarcopenia with an OR of 2.77 (95% CI 1.05–9.26) and 19.1 (95% CI 3.73–98), respectively^25^. This may be partly due to an overlap between the criteria for diagnosis of sarcopenia and frailty (handgrip strength and walking time). Additionally, the other frailty criteria (e.g., low activity and weight loss) may be associated with developing sarcopenia. From this finding, it is reasonable to screen patients for sarcopenia if they have prefrailty condition.

In our study, we found that obesity was a protective factor against sarcopenia [OR 0.35 (95% CI 0.11–1.05, *p* = 0.062)]. Several studies have shown that high BMI has protective effects against sarcopenia^26–28^. In previous study, BMI was positively correlated with muscle mass (aged-adjusted Pearson’s correlation coefficient [*r*] = 0.578, *p* < 0.001) and grip strength (age-adjusted *r* = 0.033, *p* < 0.05) which are components of sarcopenia^26^. In contrast, malnutrition tended to be a risk factor for developing sarcopenia [OR 2.16 (95% CI 0.93–4.95, *p* = 0.07)]. Malnutrition indices and sarcopenia in community-dwelling elderly are poorly investigated, and the result is still controversial. One study conducted in a geriatric outpatient population showed that the parameters for malnutrition, including the Short Nutritional Assessment Questionnaire (SNAQ), loss of appetite, unintentional weight loss, and BMI < 22 kg/m^2^, were not consistently associated with diagnostic measures of sarcopenia^29^, while other studies demonstrated that malnutrition diagnosed by the MNA, the Global Leadership Initiative of Malnutrition (GLIM), and the European Society of Clinical Nutrition and Metabolism (ESPEN) criteria were a significant risk factor for sarcopenia^30,31^. The non-statistically significant result for BMI and malnutrition in our study may be attributable to the small sample size, which could have the effect of underpowering the study.

There was no difference in the prevalence of sarcopenia between males and females in our study. The data regarding sex as a risk factor for sarcopenia remain controversial. Sarcopenia more commonly occurred in males than females in some studies^13,32–34^. Previous study showed that muscle mass deterioration in elderly males was faster than elderly females^35^. This may be due to a significant decrease in testosterone and insulin-like growth factor-1 with aging in elderly males^36^, while muscle mass in elderly females clearly reduces only during early menopause because of a significant decrease in estrogen^37^. Other studies have found that sarcopenia was more common in females than males^38,39^ or was comparable between both sexes^13,40^. The difference in ethnicity and diagnostic criteria may explain these inconsistent results.

In this study, the protein intake less than the Recommended Dietary Allowance (RDA) of 0.8 g/kg/day was not associated with sarcopenia. Protein intake above the RDA (> 1 g/kg/day), as recommended for the geriatric population, Bauer et al.^41^ was not a protective factor against sarcopenia. Previous study demonstrated that protein intake was inversely associated with muscle mass loss in the elderly of which the highest quintile of intake lost 40% less lean mass than those in the lowest intake group^42^. Higher protein intake was associated with better grip strength in participants from the Framingham Offspring cohort^43^. Nevertheless, in another study, the protein intake was not significantly associated with sarcopenia^26^. In addition, the role of protein supplementation for prevention of sarcopenia is still being debated^19,44^. It is possible that both type and amount of protein consumed may have contributed to the different results seen in the studies. Quality of protein is one of the important factors that can affect muscle protein anabolism^45^. Future research evaluating both quantity and quality of protein intake is required.

To the best of our knowledge and based on our review of the literature, this is the first study that reported the prevalence and risk factors of primary sarcopenia evaluated by the AWGS criteria in outpatient elderly. Moreover, we are the first to compare the prevalence of possible sarcopenia, sarcopenia, and severe sarcopenia as per the 2014 AWGS criteria to the 2019 AWGS criteria. This study provides additional information about prevalence and risk factors associated with this important geriatric syndrome. As with any study, there are some limitations in our study. First, participants in this study were recruited from the outpatient clinic at one hospital in Thailand and they were relatively healthy because primary sarcopenia was focused. Therefore, findings may not be generalized to those in other settings. Second, female predominant in this study may affect the prevalence and risk factors for sarcopenia and may limit generalizability of the study. Third, approximately 70% of the patients in this study had protein intake lower than the RDA. Even though the validated tool (3-day food record) was used, protein intake may be lower than the actual intake because of incomplete dietary record^46^. Forth and last, the cross-sectional design may limit the ability to define the cause and effect between the risk factors and sarcopenia. Additionally, some factors (e.g. protein intake and physical activity) may change over a period of time.

## Conclusion

In conclusion, after excluding possible causes of secondary sarcopenia, we found that 10% of the Thai community-dwelling outpatient elderly had primary sarcopenia by either the 2014 or 2019 AWGS criteria. The significant risk factors for sarcopenia were increased age, prefrailty, and low physical activity. Thus, primary sarcopenia should be evaluated in community-dwelling outpatient elderly, especially in patients with these risk factors.
